# Integrating Colon Cancer Microarray Data: Associating Locus-Specific Methylation Groups to Gene Expression-Based Classifications

**DOI:** 10.3390/microarrays4040630

**Published:** 2015-11-23

**Authors:** Ana Barat, Heather J. Ruskin, Annette T. Byrne, Jochen H. M. Prehn

**Affiliations:** 1 Centre for Systems Medicine and Department of Physiology & Medical Physics, Royal College of Surgeons in Ireland, 123 Saint Stephen’s Green, Dublin 2, D02 YN77 Ireland; E-Mails: annettebyrne@rcsi.ie (A.T.B.); prehn@rcsi.ie (J.H.M.P.); 2 Center for Scientific Computing and Complex Systems Modelling, School of Computing, Dublin City University, Collins Avenue, Dublin 9, Ireland; E-Mail: hruskin@computing.dcu.ie

**Keywords:** colorectal cancer, gene expression, locus specific methylation, colorectal cancer subtypes, microarrays, data integration

## Abstract

Recently, considerable attention has been paid to gene expression-based classifications of colorectal cancers (CRC) and their association with patient prognosis. In addition to changes in gene expression, abnormal DNA-methylation is known to play an important role in cancer onset and development, and colon cancer is no exception to this rule. Large-scale technologies, such as methylation microarray assays and specific sequencing of methylated DNA, have been used to determine whole genome profiles of CpG island methylation in tissue samples. In this article, publicly available microarray-based gene expression and methylation data sets are used to characterize expression subtypes with respect to locus-specific methylation. A major objective was to determine whether integration of these data types improves previously characterized subtypes, or provides evidence for additional subtypes. We used unsupervised clustering techniques to determine methylation-based subgroups, which are subsequently annotated with three published expression-based classifications, comprising from three to six subtypes. Our results showed that, while methylation profiles provide a further basis for segregation of certain (Inflammatory and Goblet-like) finer-grained expression-based subtypes, they also suggest that other finer-grained subtypes are not distinctive and can be considered as a single subtype.

## 1. Introduction

Cancer molecular subtyping (describing cancer subtypes) is important not least because of its potential relevance to choice of treatment [1,2]. In the case of Colon Cancer, classifications of colorectal cancers (CRC) can be subdivided into 3–6 subtypes, based on *gene expression* *profiles* [1,2,3,4]. Alternatively, CRC has been shown to divide into several subgroups according to *methylation profiles* [5]. The aim of this study is thus to establish whether there is a correlation between gene expression-based subtypes and locus specific methylation-based subgroups. Three matched expression and methylation data sets were used, in order to investigate if correlation exists between the expression and methylation subtypes. Details on the data sets are given in the next section, while the background to recent classification efforts is described below.

Efforts to classify colon cancer types according to gene expression profiles are not new: supervised approaches have been used to derive signatures related to outcomes such as recurrence, metastasis and overall survival with moderate success [6], while semi-supervised approaches exist to refine outcome prediction on (stage-related) subsets of patients [7]. More recent unsupervised efforts classify CRC into inherent molecular subtypes, which can subsequently be correlated to prognosis [1,2]. Isella *et al.* [4] have recently assessed combined information from three concurrently published independent studies (De Sousa E. Melo *et al.* [1], Sadanandam *et al.* [2], Marisa *et al.* [3]), which describe CRC classification systems (subtyping schemes), based on gene expression. Although not the primary objective of their own work, Isella and co-authors proposed a consensus classification system, based on application of the previous classifications (CCS—3 subtypes [1], CRC-Assigner—5 subtypes [2] and CCMS—6 subtypes [3], respectively) from the three studies, to the TCGA colon and rectal cancer [8] RNA-seq data [4]. Three groups are distinguished by this consensus combination of the 3 classifiers; these are: (i) the Goblet/Inflammatory group (combining the CCS2 subtype [1], the Goblet-like and Inflammatory subtypes from CRCA [2], and also C2 and C3 subtypes from the CCMS system [3]); (ii) the TA/Enterocyte group (combining the CCS1 subtype [1], the Transit Amplification (TA) and Enterocyte from CRCA [2] and also C1, C5 and C6 from CCMS [3]); and (iii) the stem/serrated/mesenchymal (SSM) group, which unites the CCS3 [1], Stem-like subtype from CRCA [2] and the EMT-associated [9,10] C4 from CCMS [3] (see Table 1). Locus-specific methylation has been long known to have variable profile across colorectal cancers, and methylation-based groups have also been identified. CIMP-status [11] detection, determined on the basis of the locus-specific methylation of (several small available panels of genes (<10 typically)) permits several methylation profiles to be distinguished [12,13]. Unsupervised clustering methylation profiles, according to data on whole-genome methylation profiling, enables refinement of the CIMP-based groups: 4 methylation-based subgroups have been identified by Hinoue *et al.* [4]. While the expression-based subtypes have been correlated with CIMP-status [1,3], the aim here was to examine the association of expression-based subtypes with whole genome-based methylation groups. In order to do so, we mapped the expression-based subtypes of Isella *et al.* [4] onto available TCGA colon and rectal cancer methylation data. Of particular interest were the questions as to whether methylation profiles can be used to refine the definition of these three expression-based type categories, ((i)–(iii)), for example by justifying the existence of the finer grained subtypes defined in CRC-Assigner [2] and CCMS [3], or to deliver new composite subtypes, thus permitting a more precise classification of molecular subtypes.

**Table 1:** Abbreviations used for the various expression-based subtypes as well as the rules for consensus subtype computation. Note that equivalent subtypes are similar but not equal.

CCS [1]	CRCA [2]	CCMS [3]	Consensus [4])	Rule for Consensus Subtype Based on CCS, CRCA and CCMS Reconciliation [4]
CCS2	Goblet-like (CRCA2)	C3 (CCMS3)	Goblet/Inflammatory	At least 2 of the assessed classifiers give equivalent subtypes.
	Inflammatory (CRCA1)	C2 (CCMS2)		
CCS1	TA (CRCA4)	C1 (CCMS1)	TA/Enterocyte	At least 2 of the assessed classifiers give equivalent subtypes.
		C5 (CCMS5)		
	Enterocyte (CRCA3)	C6 (CCMS6)		
CCS3	Stem-like (CRCA5)	C4 (CCMS4)	SSM (stem/serrated/mesenchimal)	Is computed using the union of the signature genes for for CCS3, Stem-like and C4.

## 2. Experimental Section

### 2.1. Data

Consensus subtyping information, based on RNA-seq expression data on 450 TCGA CRC samples, is available in Table S1 of Isella *et al.* [4]. These samples include both rectum adenocarcinomas (READ) and colon adenocarcinomas (COAD) cancer types. The R-package *TCGA-Assembler* [14] was used to retrieve and pre-process available methylation data from the TCGA Data Coordinating Center (DCC) for these CRC samples.

**Figure 1:**
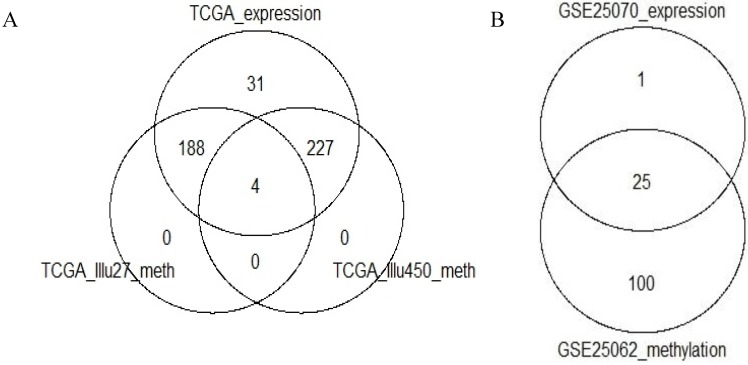
Venn diagrams showing the number of cancers for which the respective methylation and expression data types are available. (**A**) Data sets extracted from TCGA: expression-based subtyping information was available for 450 TCGA samples available from Isella *et al.* [4]. Matched methylation data, obtained with the IlluHM27 and the IlluHM450 arrays, was extracted for 192 and 231 of these 450 samples, respectively. The overlap of the two methylation data sets obtained is 4 samples; (**B**) Data sets extracted from GEO: GSE25070 contains expression data on 26 samples; GSE25062 contains IlluHM27 methylation data on 125 samples. 25 of these samples include both methylation and expression data.

Two array-based assays for measurement of DNA methylation, and available for TCGA colon and rectal cancers, have been considered: Illumina HumanMethylation27 BeadChip (IlluHM27—around 27k probes) and Illumina HumanMethylation450 BeadChip (IlluHM450—around 450k probes). 192 of the 450 considered samples with expression-based subtyping information include matched TCGA methylation data, obtained with the IlluHM27 array and 231 of the 450 samples include matched TCGA methylation data, obtained with the IlluHM450 array. Of these, 4 samples are common to both platforms, (Figure 1). Each of the two data sets, TCGA-Illu27 (192 samples) and TCGA-Illu450 (231 samples) respectively, have been pre-processed using TCGA-Assembler [14]. Pre-processing included quantile normalization of the arrays, necessary in order to provide for comparability for data obtained from distinct arrays. Batch-effect correction was not applied as the TCGA batch effect analysis, performed to assess this (see below), indicated that no correction was necessary.

In addition, methylation and expression data were retrieved from the publicly available Gene Expression Omnibus (GEO [15]): GSE25062 contains methylation data obtained with the IlluHM27 platform on 125 CRC samples, where 25 of these have array-based expression data available from the GSE25070 data set, (Figure 1). The methylation data set of 125 CRC samples, including the 25 samples with matched expression profiles, is referred to as GEO-Illu27 in what follows.

### 2.2. TCGA Batch Effect Analysis

Two approaches were used, together with existing guidelines from the literature, in order to assess the batch effects and need for correction in the two TCGA-driven data sets. Firstly, we have used Mbatch R library [16] to assess the entire COAD and READ data sets obtained with IlluHM27 and IlluHM450 platforms respectively. MBatch library permits use of hierarchical clustering and Principal Components Analysis (PCA) to identify batch effects, as well as computing metrics to quantify these: Dispersion Separability Criterion (DSC) and DSC *p*-value. DSC is a ratio of between batch dispersion *vs.* within batch dispersion. Batch effects are considered to be present if the DSC *p*-value < 0.05 *and* the DSC value high (>0.5). If *either* condition was false, batch effects are considered not significant. Secondly, we have applied hierarchical clustering and PCA to the TCGA-Illu27 and TCGA-Illu450 methylation data sets containing both COAD and READ samples each. For hierarchical clustering, we used the average linkage algorithm, (with the dissimilarity measure given by 1 − r, with r the Pearson correlation coefficient). Samples were clustered and annotated by coloured bars, with each colour representing a Batch ID, a Plate ID or a TSS (Tissue Source Site). For the PCA, methylation values of the samples were projected onto the plane, defined by the first two principal components, and were annotated once again with different colours for different batches, plates or TSS.

### 2.3. CIMP-Status Prediction

Indicative CIMP status of the TCGA tumours analysed here was determined using the principle of two gene panels, (proposed Hinoue *et al.* [5]), to identify CIMP positive and CIMP-H tumors, respectively, but with more conservative criteria for attribution, namely the requirement of *four or more* methylated targets for each gene panel instead of just three, with a *higher threshold* methylation *β*-value [17] (0.3) required for determining CIMP-H cases. The main reason for this more stringent condition is to ensure conservative assessment; the three loci only paradigm appears insufficiently precise in identification of CIMP and CIMP-H tumors in the data sets used here, (resulting in an unrealistically high number of these).

### 2.4. Clustering the DNA Methylation Data

Preprocessing steps, required before clustering the DNA methylation β-values [17] include removing the NA-masked [18] data points and probes with sequences on X and Y chromosomes (asdescribed in [8], Supplementary Material). Only probes with standard deviation of the methylation *β*-values S.D. > 0.18 (for the Illu27 data sets) and S.D. > 0.23 (for the Illu450 data set) have been retained for clustering. For genes, targeted by multiple probes, only the most variable probes have been selected. Subsequent to these processing steps, 2222, 2637 and 1136 probes remained in the TCGA-Illu27, TCGA-Illu450 and GEO-Illu27 data sets, respectively.

Two unsupervised clustering approaches were used in order to identify methylation-based tumor subgroups:

(1) the average linkage agglomerative clustering method with 1 − r, with r the Pearson correlation coefficient, dissimilarity measure (*hclust* function in the R software) and
(2) the recursively partitioned mixture model (RPMM), (implemented in the R software RPMM package) [19].

The *heatmaps* for graphical representation of the DNA methylation β-values were generated using a modification of the R *heatmap* function. To determine cluster stability, bootstrap resampling (of the cancer samples) was performed using the *clusterboot* function from the *fpc* R package [20,21]. Alignment of most similar resampled clusters to original clusters was achieved using Jaccard similarities [22] of the latter, [20,21]. For Jaccard similarity values ≤ 0.5, the number of items which differ between the original cluster and the most similar in the resampled data is *at least as large* as the number for which these coincide [20], where such clusters are considered unstable or “dissolved”. A valid and stable cluster should yield a mean Jaccard similarity value ≥ 0.75; while clusters with Jaccard values between 0.6 and 0.75 provide some evidence of patterns in the data, they exhibit fuzziness in terms of specific attribution of cluster elements. In general, Jaccard values < 0.6 indicate dispersed or ill-defined clusters [20].

**Figure 2:**
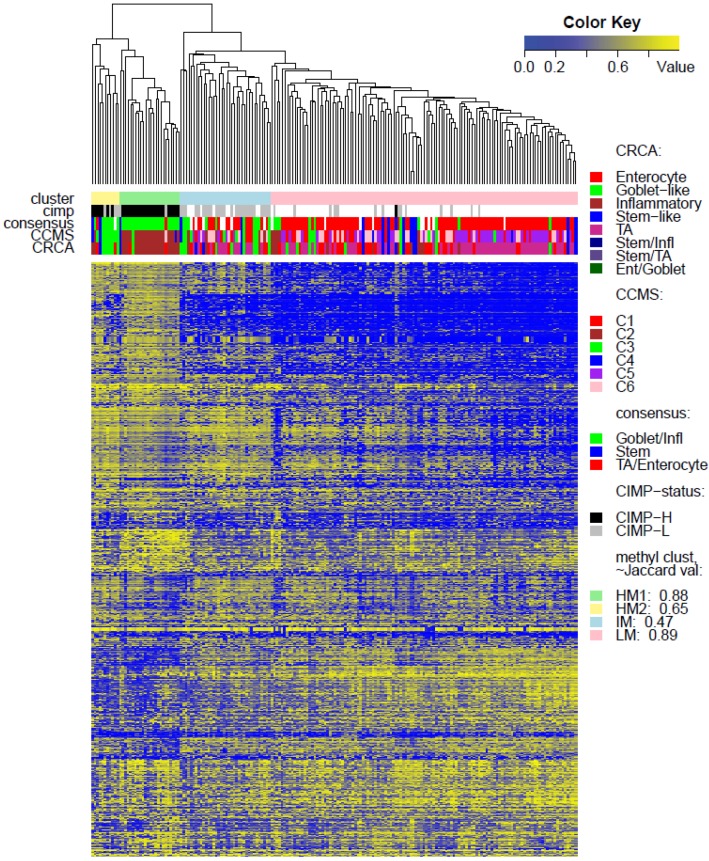
Heatmap of methylation values for data set *TCGA-Illu27*: clusters based on the average linkage agglomerative clustering method with 1 minus Pearson correlation distance measure. Annotation, by expression-based subtypes of CRCA [2], CCMS [3], and consensus [4] classifiers, is given by upper horizontal bands. For each annotated cluster, the Jaccard similarity value is given. For Jaccard ≤ 0.5, the cluster is unstable or dissolved; for 0.5 < Jaccard ≤ 0.6 the cluster is dispersed or ill-defined; for 0.6 < Jaccard < 0.75 the cluster is fuzzy but shows a pattern in the data; for Jaccard ≥ 0.75 the cluster is stable.

**Figure 3:**
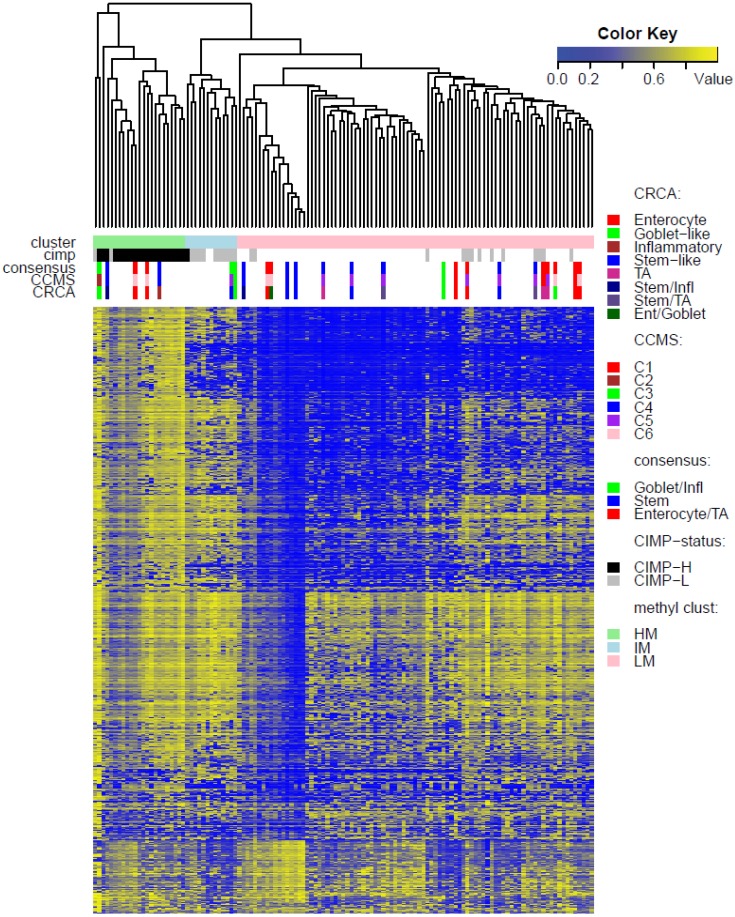
Heatmap of methylation values for data set *GEO-Illu27*: clusters based on the average linkage agglomerative clustering method with 1 minus Pearson correlation distance measure. Annotation, by expression-based subtypes of CRCA [2], CCMS [3], and consensus [4] classifiers, is given by upper horizontal bands.

**Figure 4:**
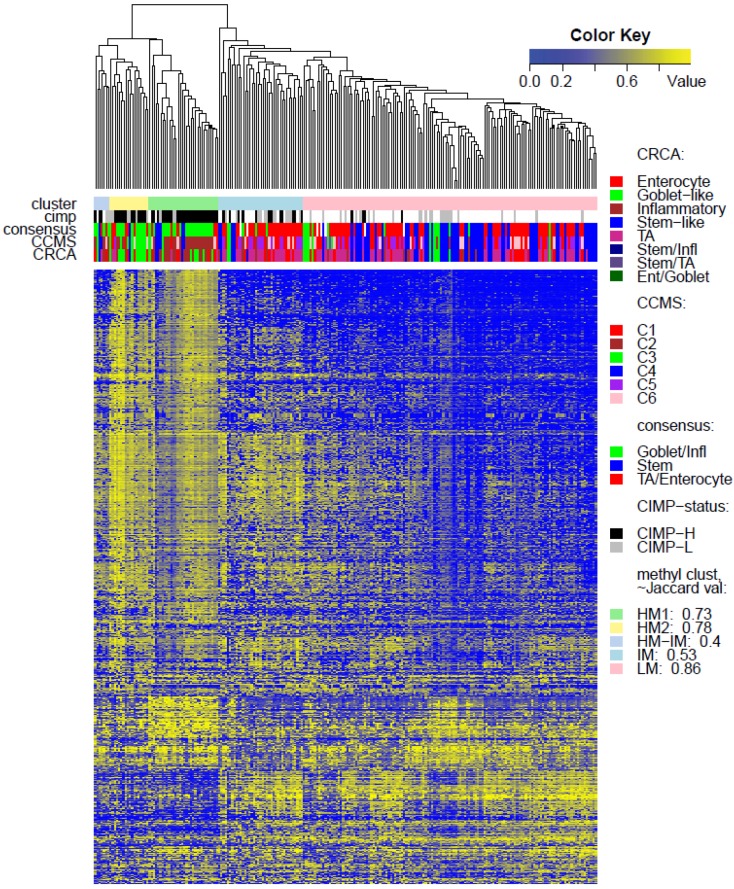
Heatmap of methylation values for data set *TCGA-Illu450*: clusters based on the average linkage agglomerative clustering method with 1 minus Pearson correlation distance measure. Annotation, by expression-based subtypes of CRCA [2], CCMS [3], and consensus [4] classifiers, is given by upper horizontal bands. For Jaccard ≤ 0.5, the cluster is unstable or dissolved; for 0.5 < Jaccard ≤ 0.6 the cluster is dispersed or ill-defined; for 0.6 < Jaccard < 0.75 the cluster is fuzzy but shows a pattern in the data; for Jaccard ≥ 0.75 the cluster is stable.

**Figure 5:**
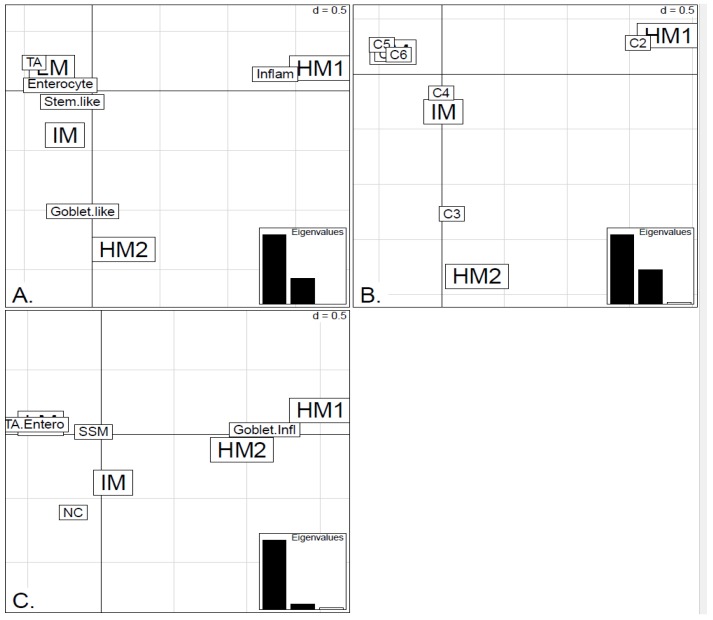
Factorial Correspondence Analysis (FCA) between the methylation clusters obtained with average linkage clustering (HM1, HM2, IM and LM) and the various expression-based subtype classifiers: (**A**) CRCA; (**B**) CCMS; (**C**) consensus in the *TCGA-Illu27* data set. Spatial proximity between two labels illustrates closeness/correspondence of the labelled modalities. For all three comparisons, the *χ^2^* independence test yielded *p*-value < 10^−15^.

Overlap between the two clustering algorithms has been assessed by computing *χ^2^* independence tests on the samples labelled with the methylation cluster classes obtained by each technique, respectively. In addition, Factorial Correspondence Analysis has been performed in order to graphically represent the correspondence between the obtained methylation classes (see details in the next subsection).

The *nearest shrunken centroids* classifier [23], an enhancement of the nearest centroid classifier, has been applied to the methylation data, in order to identify the subset of genes that best characterizes the highly methylated subclusters, where these are significantly enriched for different expression subtypes. The R package *PAMR* has been used to apply the classification method.

### 2.5. Factorial Correspondence Analysis (FCA)

To represent graphically the correspondence between the various expression-based subtype classifiers and the methylation clusters obtained with average linkage clustering, contingency tables were first computed between the samples, labelled with subtypes obtained with three expression classifiers and by the methylation cluster classes, respectively. In this case, the expression subtypes and the methylation clusters behave like factors with multiple modalities, with every sample having only one modality per factor. Subsequently, FCA was performed for the contingency tables. FCA is a dimension reduction technique where the multiple modalities of the 2 factors in a contingency table are represented in a 2D space. Mathematically, FCA’s principle is close to computing a *χ^2^* independence test between two factors, with the advantage of modality correspondence visualization. Balloonplots, a direct graphical representation of contingency tables, was a different way to represent correspondence between factor modalities. The R libraries *ade4* and *gplots* were used for FCA and Balloonplots, respectively.

### 2.6. Statistics

In order to assess whether a subtype is specific to a cluster, contingency tables have been computed and frequencies have been compared using the Fisher Exact Test. Unless otherwise specified, the *p*-values for estimation and testing presented here refer to the Fisher Exact Test.

## 3. Results and Discussion

The work of Marisa *et al.* [3] investigated association of the CCMS expression subtypes to CIMP status, and in order to expand this by considering expression-based subtype association with whole genome locus-specific methylation profiles, three clustered methylation data sets: TCGA-Illu27, TCGA-Illu450 and GEO-Illu27 (see Section 2), were annotated with expression-based subtypes.

Two whole genome methylation data sets (TCGA-Illu27 and TCGA-Illu450) have been extracted from TCGA and pre-processed as described in Section 2. No data set was initially corrected for batch effects. Figures S1–S4 show the PCA and hierarchical clustering according to the TCGA Batch ID, Plate ID and TSS for the TCGA-Illu27 and TCGA-Illu450 data sets, respectively. For TCGA-Illu27, only one batch (29), is found distinct from the other batches. However, Cancer Genome Atlas Network argues that the differences observed for this batch are biological rather than technical, (Supplementary Material from [8]), not least as it consists entirely of MSI/CIMP subtype samples, (Supplementary Material [8]). No other batches have been found to have important effects. In addition, Table S1 gives the Dispersion Separability Criterion (DSC) values (computed with respect to Batch ID) obtained for each complete TCGA COAD and READ data set, assessed with IlluHM27 and IlluHM450 platforms respectively. In accordance with our PCA and hierarchical clustering results, all four complete sets (COAD and READ on each of the IlluHM27 and IlluHM450 platforms respectively) have DSC < 0.5, indicating unimportant batch effects. The largest DSC has been obtained, as expected, for the COAD IlluHM27 set, which contains batch 29. Taken together, these results indicate that batch effects correction was not necessary for the TCGA-based data sets. After filtering the methylation values according to methylation value standard deviation cutoffs (see Section 2), 2222, 2637 and 1136 probes remained in the TCGA-Illu27, TCGA-Illu450 and GEO-Illu27 data sets, respectively. The intersection between the remaining genes in the two TCGA-based data sets was of 900 genes.

Two unsupervised clustering methods (average linkage agglomeration clustering and RPMM), were applied to the methylation values for each data set. Both methods distinguished three main clusters, (Figure 2, Figure 3 and Figure 4 for average linkage agglomeration and Figures S5 and S6 for RPMM clustering): these consisted of a highly methylated (HM) cluster, (predominantly CIMP-H, featuring two rather distinct sub-clusters HM1 and HM2 [24] for the two TCGA data sets), an intermediately methylated (IM) cluster (including both CIMP-H and CIMP-L) and a large cluster with both lower and rarer locus-specific methylation (LM), (predominantly non-CIMP). The overlaps between the clusters obtained with the two different algorithms, for both TCGA data sets), are very good (*χ^2^* independence tests yielded *p*-values < 10^−15^). The correspondence between the labels of the methylation classes obtained with the two clustering algorithms, respectively, are illustrated by Factorial Correspondence Analysis in Figure S7 for the two TCGA-based data sets.

In agreement with previous studies [1,3] which found:

(a) CCS2 from [1] and both C2 and C3 (consensus Goblet/Inflammatory in [3]) to be frequently CIMP positive (CIMP+)
(b) CCS1 from [1] and C1, C5 and C6 (consensus TA/Enterocyte in [3]) to be more frequently CIMP negative (CIMP−),

strong associations were observed for both TCGA data sets, between the consensus Goblet/Inflammatory expression subtypes and the highly methylated (HM) cluster (*p*-value < 10^−11^, Fisher Exact Test), as well as between the consensus Enterocyte/TA expression subtypes and the lower methylated (LM) cluster (*p*-value < 10^−11^).

**Figure 6:**
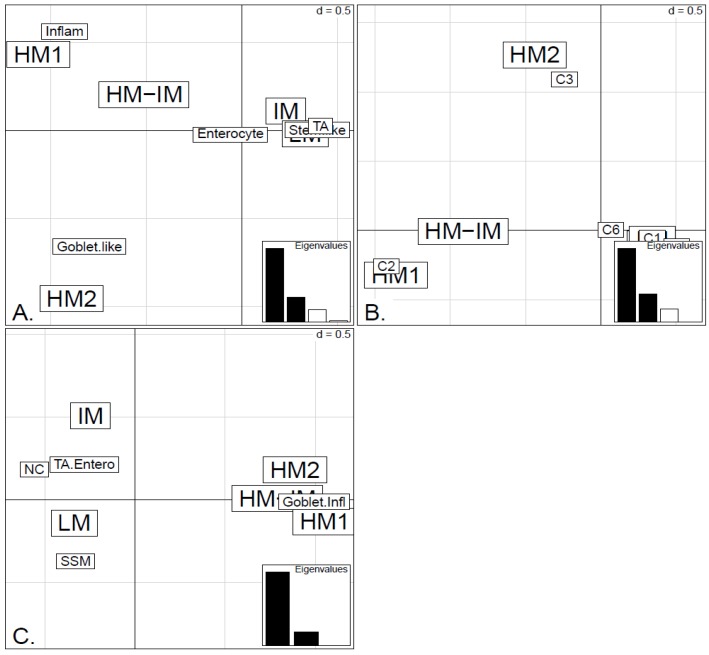
Factorial Correspondence Analysis (FCA) between the methylation clusters obtained with average linkage clustering (HM1, HM2, IM and LM) and the various expression-based subtype classifiers: (**A**) CRCA; (**B**) CCMS; (**C**) consensus in the *TCGA-Illu450* data set. Spatial proximity between two labels illustrates closeness/correspondence of the labelled modalities. For all three comparisons, the *χ^2^* independence test yielded *p*-value < 10^−12^.

**Figure 7:**
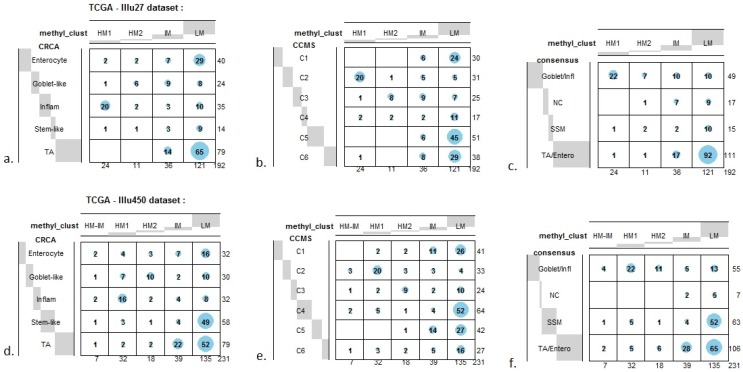
Bubbleplots - graphical representations of contingency tables for expression-based subtype classifiers (CRCA, CCMS, consensus respectively) and methylation based clusters, obtained with hierarchical clustering (average-linkage agglomerative method), for the *TCGA-Illu27* (a, b, c) and *TCGA-Illu450* (d, e, f) data sets. The highlighted areas indicate proportional correspondence between factor modalities. For the contingency tables with consensus subtyping scheme, NC refers to those samples that do not have a consensus classification.

In order to illustrate correspondence between the obtained methylation clusters and the various expression-based subtypes (CRCA, CCMS and consensus), Factorial Correspondence Analysis (FCA) and Balloonplots were applied. The associations between expression subtypes and HM, IM and LM clusters are very well illustrated by spatial proximity in the correspondence analysis, Figure 5 and Figure 6 and are highlighted on the contingency tables representations by Balloonplots (Figure 7c,f).

While Marisa *et al.* [3] also found C4 (consensus SSM) to be associated with CIMP+, SSM was not associated with any of the methylation clusters in TCGA-Illu27 (Figure 2). This is possibly due to the fact that SSM represents only 8% of this data set. It was, however, significantly associated with LM cluster values in GEO-Illu27 (*p*-value = 0.087, Fisher Exact Test, and more conservatively, *p*-value = 0.048, Barnard’s Test, Figure 3) and also in TCGA-Illu450 (*p*-value < 10^−5^, Fisher Exact Test, Figure 4 and Figure 6C, Figure S5). In both GEO-Illu27 and TCGA-Illu450 data sets, SSM represented a larger fraction of all data: 40% and 27% respectively, compared to the TCGA-Illu-27 case. Given the fact that Stem-like samples have very distinctive clinical features (especially high relapse rate in untreated cases and increased sensitivity to chemotherapy in metastatic settings [2]), a distinctive methylation profile for these samples may be expected, too. However, this is not the case, most SSM being distributed across the LM cluster. Moving from HM to LM, the preponderance of stem-like SSM becomes much higher, with the opposite true for Goblet/Inflammatory, suggesting a gradient for inclusion of these subtypes as overall methylation reduces or increases, with clearly-defined bands observed in the LM sub-clusters obtained with the RPMM method, (Figure S6). Recent work [4] has indicated that the stem-like and EMT features of the SSM subtype are in fact contributions from a distinctively abundant stromal fraction of these tumors. In fact, Isella *et al.* [4] show that it may be the stromal fraction in the SSM samples that influence their clinical features. Taking these observations together offers some support for the argument that, without their stromal content, the SSM samples may fall into the category of one the remaining subtypes, as noted in [4]. The annotated methylation heatmaps here suggest that it is the low methylated profile which is enriched with samples with a high stromal fraction, *i.e.*, the SSM subtype.

As well as using the consensus subtypes defined in [4], the current analysis looked at methylation clusters annotated by the finer grained subtypes CRCA [2] and CCMS [3]. Focusing on the TCGA-Illu27 data set, results in Figure 1 and Figure 5A,B suggest that CRCA Inflammatory and CCMS C2 subtypes were not randomly distributed across the HM cluster as a whole, but are quite specific to HM1, (*p*-values < 10^−15^, for both Inflammatory and C2). Noted in this regard is the quasi co-localisation of HM1 with Inflammatory and C2 on Figure 5A,B respectively. Additionally, HM1 was the largest and most stable (Jaccard similarity value = 0.88) of the two component sub-clusters found here by agglomerative clustering: 74% of all CRCA Inflammatory and 66% of all CCMS C2 subtypes were found within this sub-cluster (with 86% of its overall composition due to the two), (Figure 7a,b and Table S2). The second largest highly-methylated sub-cluster HM2 was mainly populated by subtypes which are Goblet-like and CCMS C3 equivalent subtypes, but with these being less specific to this one sub-cluster (*p*-values < 10^−4^ for both Goblet-like and C3), (Figure 7a,b, Table S3). It is also worth noting that associations between HM2 and Goblet-like and C3 are obvious but less strong on the correspondence charts (Figure 5A,B). The associated Jaccard similarity value of 0.64 does indicate a possible pattern in the data but, as highlighted by application of bootstrapping [20,21], attribution of these samples to the specific sub-cluster was less clear-cut and other attributions are also possible.

Very similar observations apply to HM1 and HM2 in the TCGA-Illu450 data set; in this case Inflammatory/C2 were found to be specific to HM1 (*p*-values < 10^−7^), and Goblet-Like/C3 specific to HM2 (*p*-values < 10^−5^), (Figure 6A,B and Figure 7d,e, Tables S2 and S3). These results suggested that CIMP+ Inflammatory/C2 are characterized by quite distinct methylation profiles as opposed to CIMP+ Goblet-like/C3, which are subject to more heterogeneous methylation. With RPMM clustering, a predominantly Inflammatory/C2 HM cluster was obtained also for the TCGA-Illu27 data set. The CIMP+ Goblet-like/C3 samples are distributed between both HM and IM clusters, (Figure S5), reinforcing the observation that there is considerable methylation heterogeneity in the Goblet-like/C3 classification. For TCGA-Illu450, the RPMM method yielded two HM clusters, which are labelled HM1 and HM2, (Figure S6) and share >65% members with the HM clusters obtained by agglomerative clustering.

The fact that Inflammatory/C2 clearly segregate from Goblet-like/C3 subtypes when methylation profiles are examined, suggests that it may be interesting to consider these subtypes separately, instead of combined or paired in a single subtype. Interestingly, Inflammatory and Goblet-like subtypes seem to behave differently in terms of treatment response association. Sadanandam *et al.* [2] evaluated disease-free survival (DFS) in both untreated and treated (adjuvant chemotherapy or radiotherapy) patients. In untreated patients, Goblet-like showed a good prognosis, while Inflammatory subtypes had intermediate DFS. In treated patients, adjuvant chemotherapy or radiotherapy was detrimental for Goblet-like subtypes but made no difference for Inflammatory subtypes [2]. These authors have also examined the possibility that the subtypes show different responses to FOLFIRI, a chemotherapy regimen used in first-line treatment of *metastatic* CRC, by deriving a FOLFIRI response signature for the analysed samples where actual FOLFIRI response was not available [2,25]. Inflammatory subtype appears to be the second subtype the most associated to FOLFIRI response signature (after Stem-like subtype), while Goblet-like subtype is less associated to FOLFIRI response signature [2].

In order to find genes, with methylation distinctive to HM1 or HM2, the shrunken nearest centroids method [23] was applied to methylation data of identified HM1 and HM2 samples, for each of the TCGA-Illu27 and TCGA-Illu450 data sets. With shrinkage thresholds chosen to yield cross-validation error-rates (<0.05), a total, respectively, of 106 and 129 genes, which best characterized the HM classes for the two data sets, were obtained. Of these two gene lists, 7 genes were found to be common for both data sets and 6 of these (GDF5, SOX8, KRT20, SMOC1, OLFM4 and SLC6A3) had same sign shrunken centroids for both classes.

In contrast to Goblet and Inflammatory subtypes, TA and Enterocyte subtypes of the CRCA classifier and C1, C5 and C6 subtypes of the CCMS classifier seemed to be both heterogeneous and dispersed across IM and, in particular, LM clusters. It should be noted that, for TCGA-Illu27, the far right LM sub-cluster comprising 48 samples, (Figure 2), was significantly associated with TA and C5 subtypes (*p*-value < 10^−3^) and contains the lowest methylated samples of this data set. A TA/C5 sub-cluster, such as this, was not observed for agglomerative clustering of the TCGA-Illu450 data set, (Figure 4). From the methylation data viewpoint at least, the primary interest might be to consider TA and Enterocyte/C1, C5 and C6 subtypes under a consensus TA/Enterocyte signature, such as the one suggested in [4]. In terms of clinical features, Sadanandam *et al.* [2] reported some differences for the TA and Enterocyte subtypes, the main one being that untreated TA showed better prognosis than untreated Enterocyte. The differences found in terms of response to treatments were less important than those found for Goblet-like and Inflammatory subtypes, ([2] and Supplementary Material therein).

Finally, tumors with intermediate methylation (IM) profiles appeared to be the most heterogeneous with respect to the expression-based subtypes, (exhibiting a mixture of Goblet-like/C3 and Enterocyte/C1, C5, C6), and also to methylation profiles (“unstable, dissolved” and “dispersed” IM clusters, with Jaccard similarity values of 0.48 and 0.53 obtained for the TCGA-Illu27 and TCGA-Illu450 data sets, respectively).

## 4. Conclusions

In this analysis, whole-genome *methylation profiles* from three publicly available data sets have been used to characterize three independent gene expression-based colon cancer classifiers, which separate CRC into between three and six groups. Our analysis indicates that four of the expression-based subtypes are most distinctive: namely consensus SSM [4], consensus TA/Enterocyte [4], Goblet-like [2]/C2 [3] combined and Inflammatory [2]/C3 [3] combined. The Goblet-like/C2 and Inflammatory/C3 subtypes segregated into two groups by *methylation*, (where this is characteristically high compared to other subtypes), and hence may be considered as two close, but separate, subtypes. In contrast, neither Enterocyte and TA (CRCA) nor C1, C5, C6 (CCMS), were distinctive in terms of locus-specific methylation and can be considered together or separately depending on the aim of the study. We also find the SSM consensus subtype, as well as the equivalent Stem-like and C4, to be correlated with low methylation but only somewhat distinct from TA/Enterocyte. Finally, we find that the Intermediately Methylated group contained a mixture of all expression subtypes indicating that gene expression-methylation association is indeterminate in this case. Our findings demonstrate that integration and combined analysis of microarray-based gene expression and methylation data sets offers potential for a better description of the CRC subtypes.

## Supplementary Files

Supplementary File 1

## Notes Added in Proof

Following acceptance of this manuscript for publication (October 2015), Guinney *et al.* [26], whose objective was to resolve inconsistencies among the reported gene-expression-based classifications, revealed 4 CRC consensus molecular subtypes (CMS1-4) obtained from bringing together 4151 patients from 18 CRC data sets. In particular, CMS1 covers almost all patients labelled Inflammatory [2] and almost all C2 [3]. Likewise, CMS3 covers almost all Goblet-like [2] and C3 [3]. Our current study is in agreement with this work, suggesting that Goblet-like/C2 and Inflammatory/C3 are distinctive based on their methylation profile. In addition, Guinney *et al.* presented CMS2 which covers both TA and Enterocyte [2] and C1, C5 and C6 [3] respectively, again in agreement with our methylation-based results. Table 2 gives the correspondence between the classifiers discussed in this paper and the new more robust CMS classifier [26].

**Table 2:** Abbreviations used for the various expression-based subtyping classifiers used in this paper as well as the newly published consensus classifier CMS [26]. Subtypes on the same line tend to label the same samples, but there are samples for which consensus subtype cannot be computed, [26].

CCS [1]	CRCA [2]	CCMS [3]	Consensus [4]	Consensus [26]
CCS2	Goblet-like (CRCA2)	C3 (CCMS3)	Goblet/Inflammatory	CMS3—metabolic (abt 13%, epithelial and methabolic dysregulation)
	Inflammatory (CRCA1)	C2 (CCMS2)		CMS1—MSI immune (abt 14%, hypermutated, MSI, strong immune activation)
CCS1	TA (CRCA4)	C1 (CCMS1)	TA/Enterocyte	CMS2—canonical (abt 37%, epithelial, marked WNT and MYC signaling activation)
		C5 (CCMS5)		
	Enterocyte (CRCA3)	C6 (CCMS6)		
CCS3	Stem-like (CRCA5)	C4 (CCMS4)	SSM (stem/serrated/mesenchimal)	CMS4—mesenchymal (23%, TGF-β activation, stromal invasion, angiogenesis)
