# Supplementary material for: Integrating Colon Cancer Microarray Data: Associating Locus-Specific Methylation Groups to Gene Expression-Based Classifications
Source: Microarrays (Basel). 2015 Nov 23;4(4):630–46. doi: 10.3390/microarrays4040630 (PMC4996409; doi:10.3390/microarrays4040630)
Supplement: Supplementary File 1 [file microarrays-04-00630-s001.pdf]

Supplementary Information

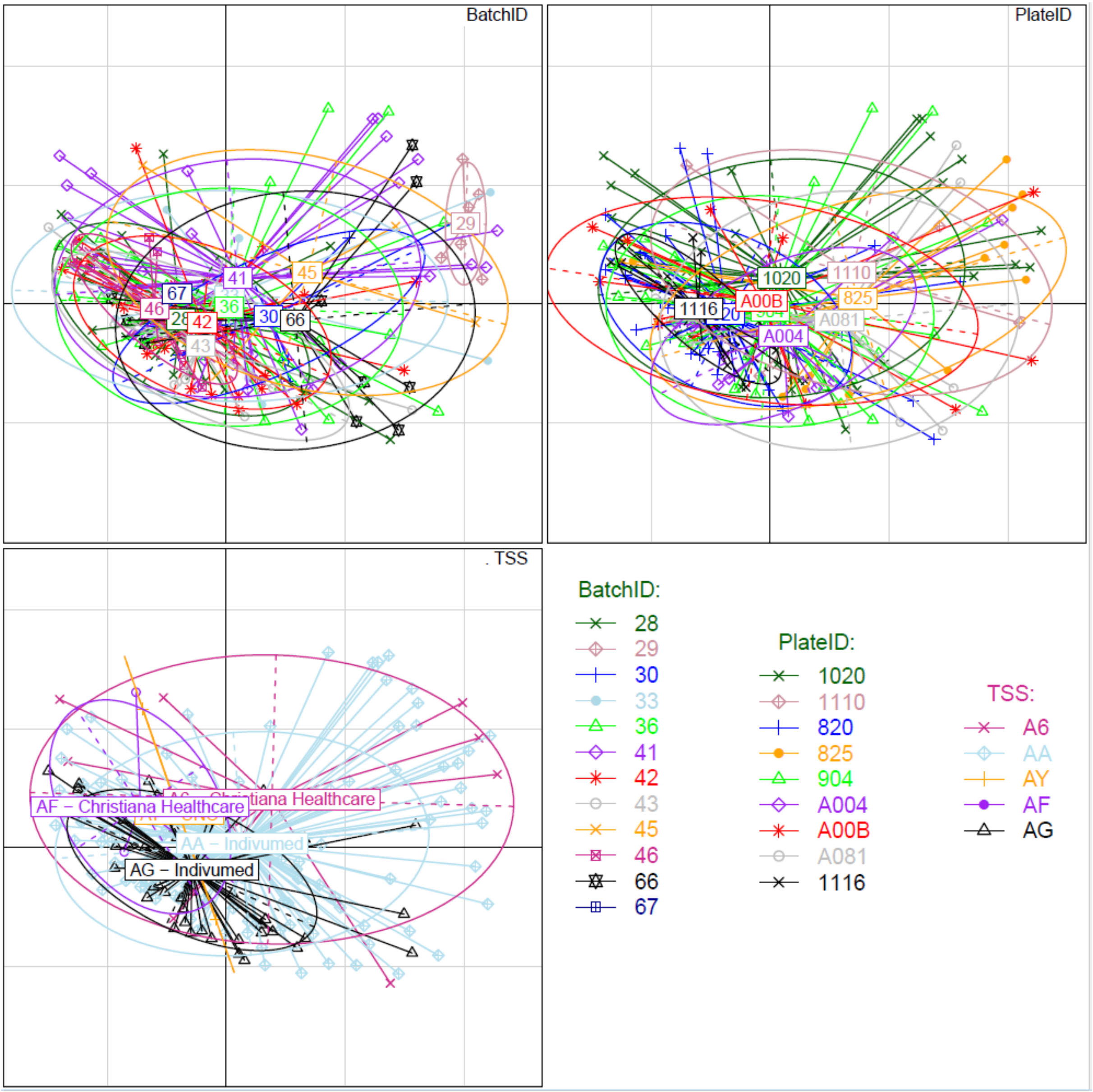

Figure S1. PCA for DNA methylation obtained with IlluHM27 BeadChip with samples connected by centroids according to Batch ID, Plate ID and TSS. The inertia ellipses of the points constituting each specific batch, plate or TSS together with the batch centroids are represented on the plan defined by the first two principal components. The centroids have been obtained by projecting on the plan the methylation mean across all samples in the batch. Each centroid is labelled by the batch, plate or TSS ID.

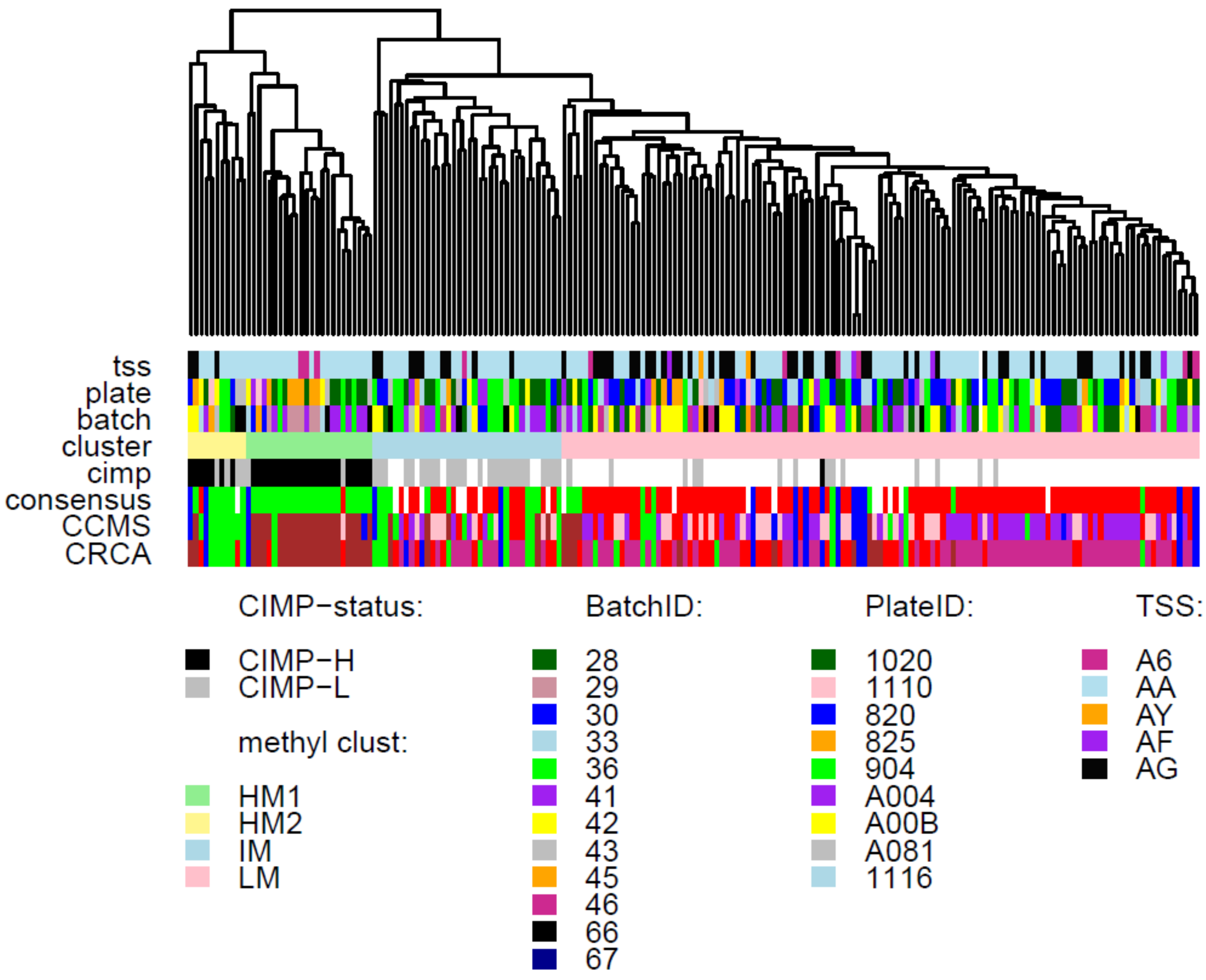

Figure S2. Hierarchical clustering plot (average-linkage agglomerative method) for DNA methylation data obtained with IlluHM27 BeadChip, with samples annotated by Batch ID, Plate ID and TSS. Colours from the unannotated horizontal bars correspond to those from Figure 2.

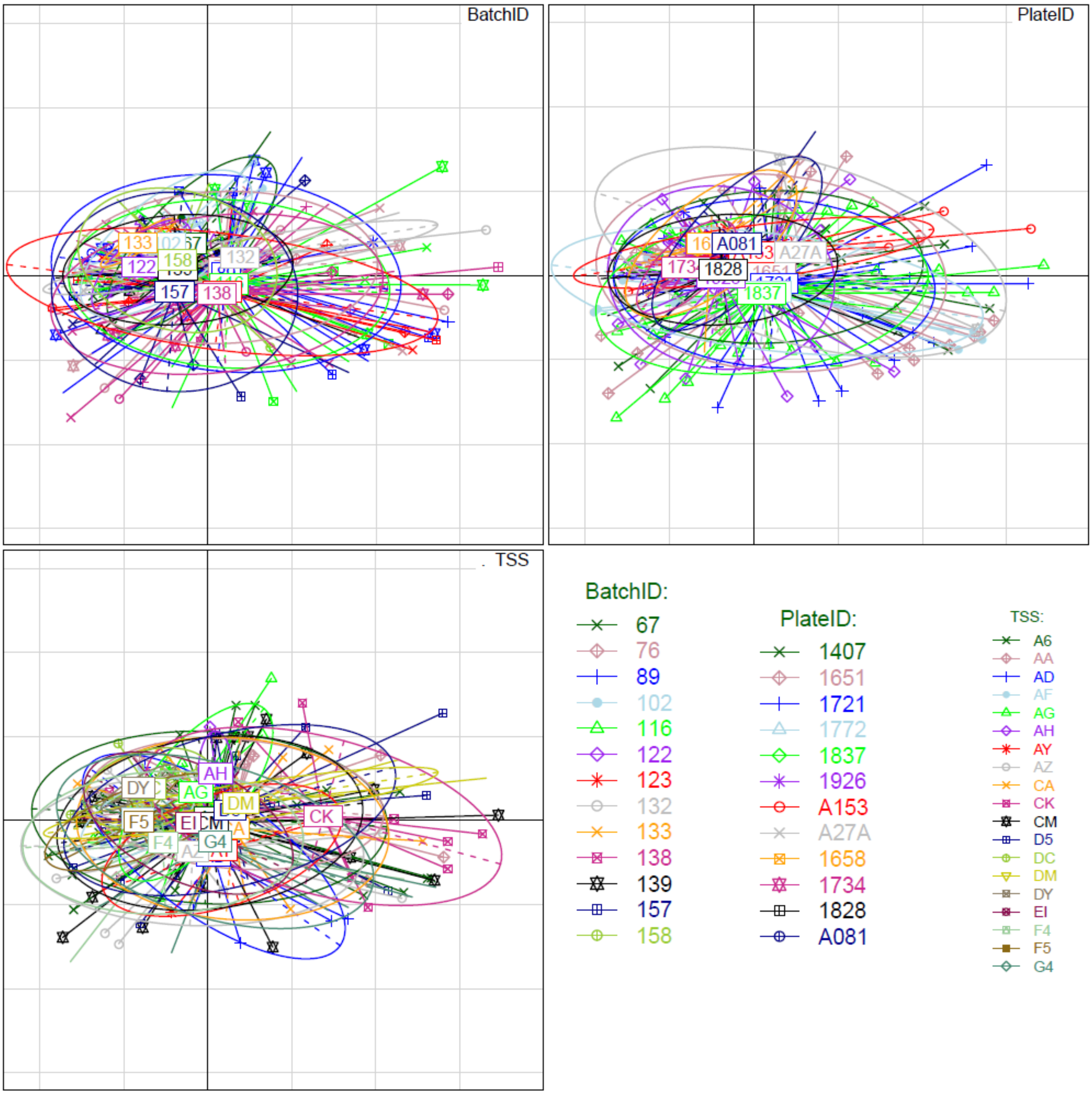

Figure S3. PCA for DNA methylation obtained with IlluHM450 BeadChip with samples connected by centroids according to Batch ID, Plate ID and TSS. The inertia ellipses of the points constituting each specific batch, plate or TSS together with the batch centroids are represented on the plan defined by the first two principal components. The centroids have been obtained by projecting on the plan the methylation mean across all samples in the batch. Each centroid is labelled by the batch, plate or TSS ID.

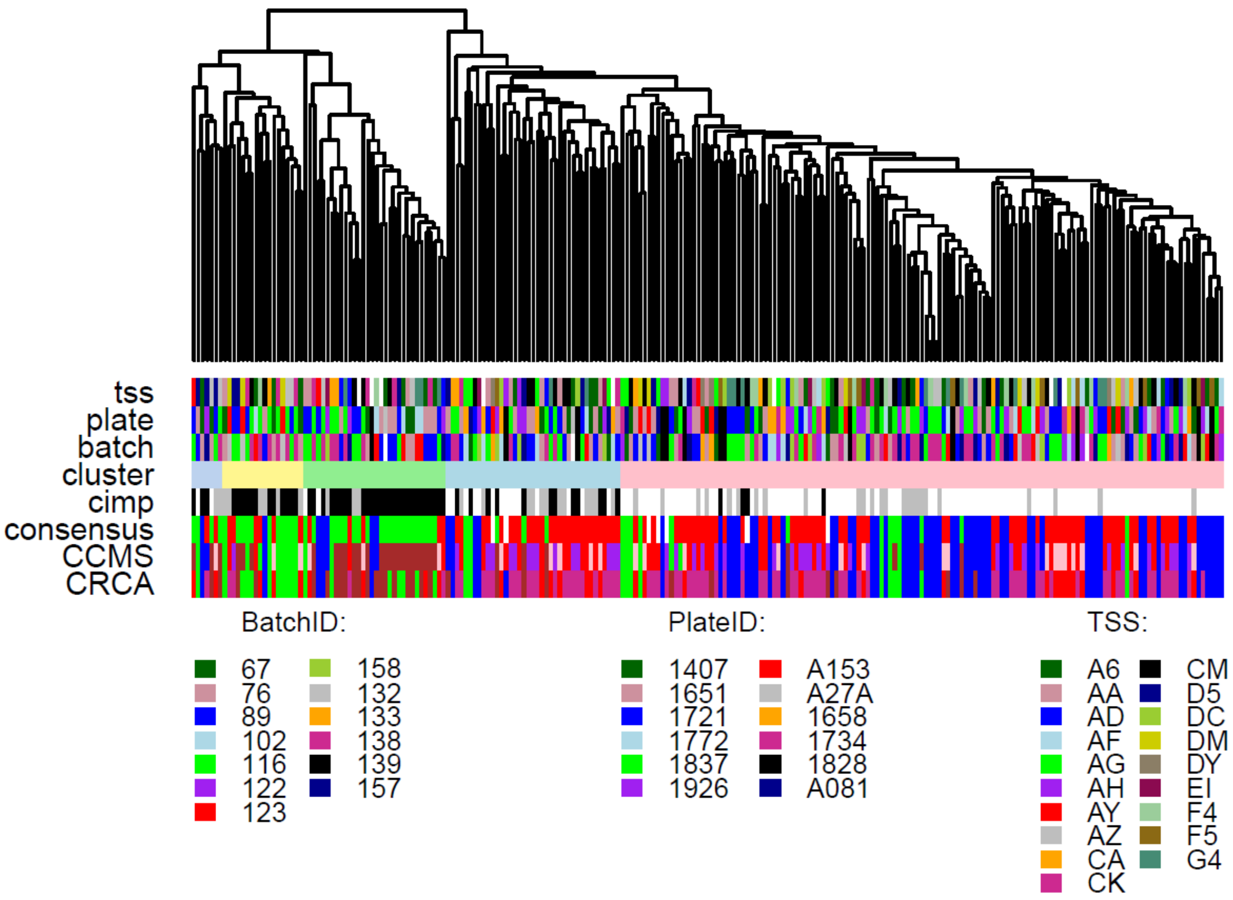

Figure S4. Hierarchical clustering plot (average linkage agglomerative method) for DNA methylation data obtained with IlluHM450 BeadChip, with samples annotated by Batch ID, Plate ID and TSS. Colours from the unannotated horizontal bars correspond to those from Figure 4.

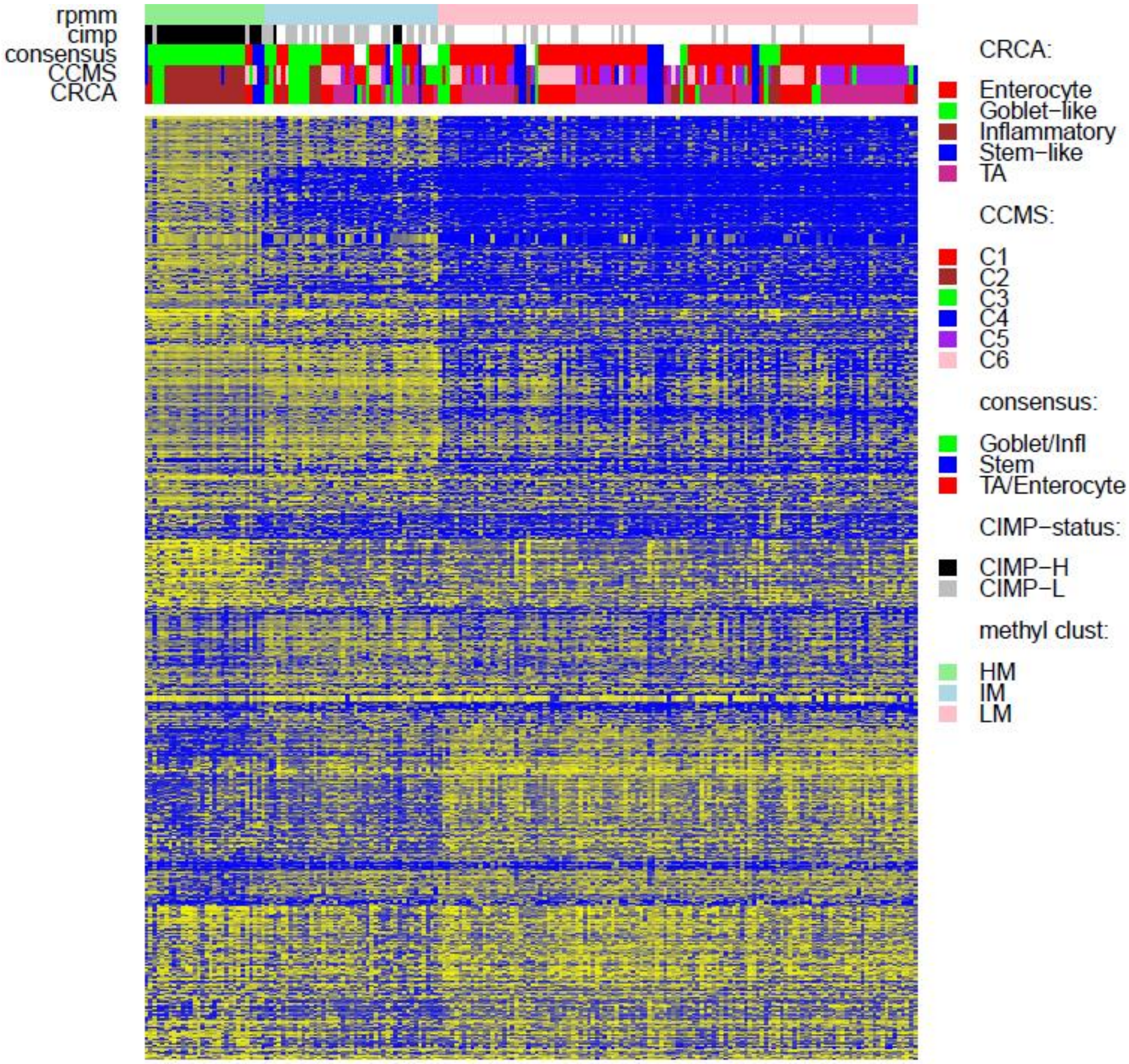

Figure S5. Heatmap of methylation values for data set TCGA-Illu27: clusters based on the recursively partitioned mixture model (RPMM) method. Annotation, by expression-based subtypes of CRCA [2], CCMS [3], and consensus [4] classifiers, is given by upper horizontal bands.

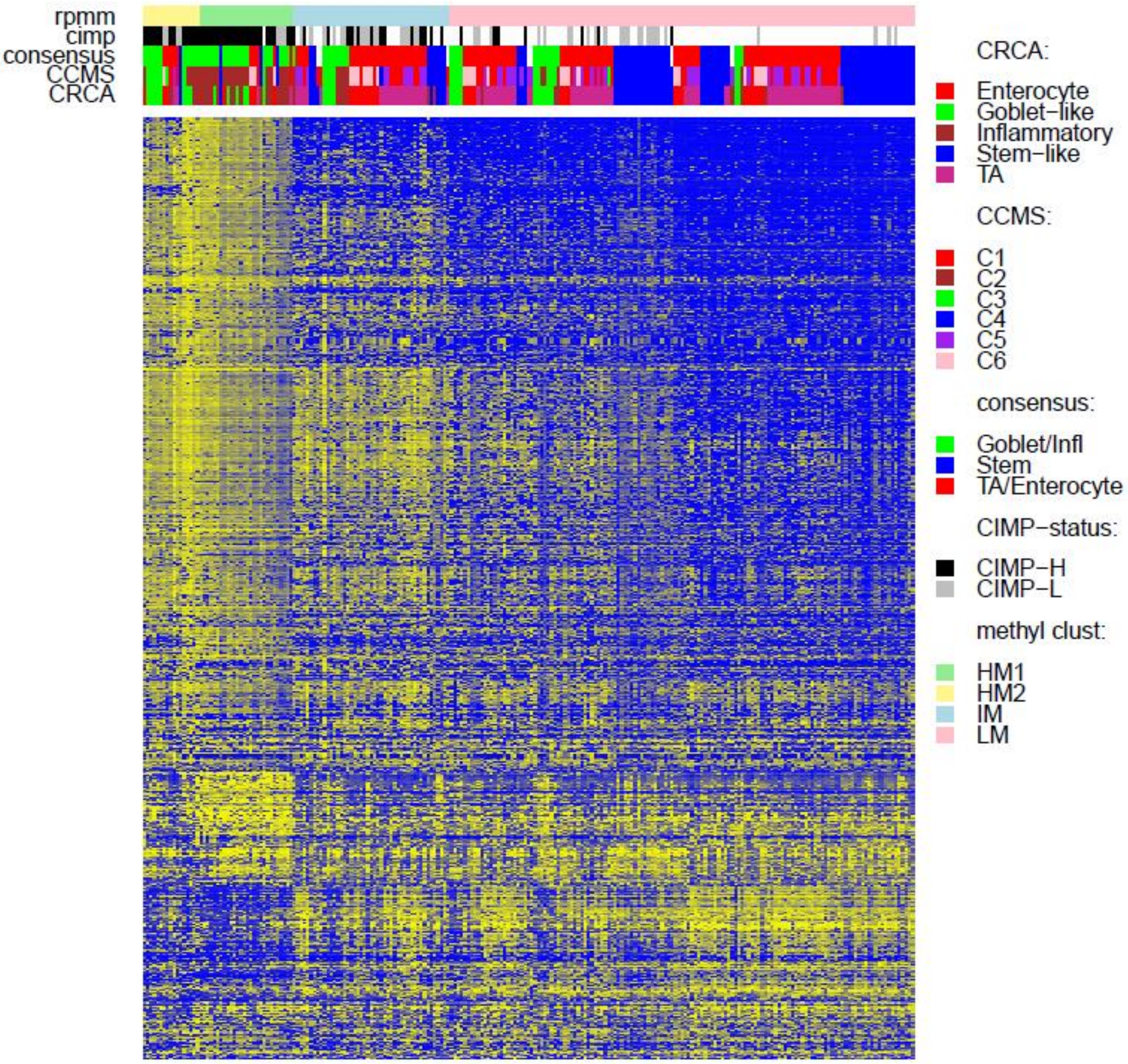

Figure S6. Heatmap of methylation values for data set TCGA-Illu450: clusters based on the recursively partitioned mixture model (RPMM) method. Annotation, by expression-based subtypes of CRCA [1], CCMS [2], and consensus [3] classifiers, is given by upper horizontal bands.

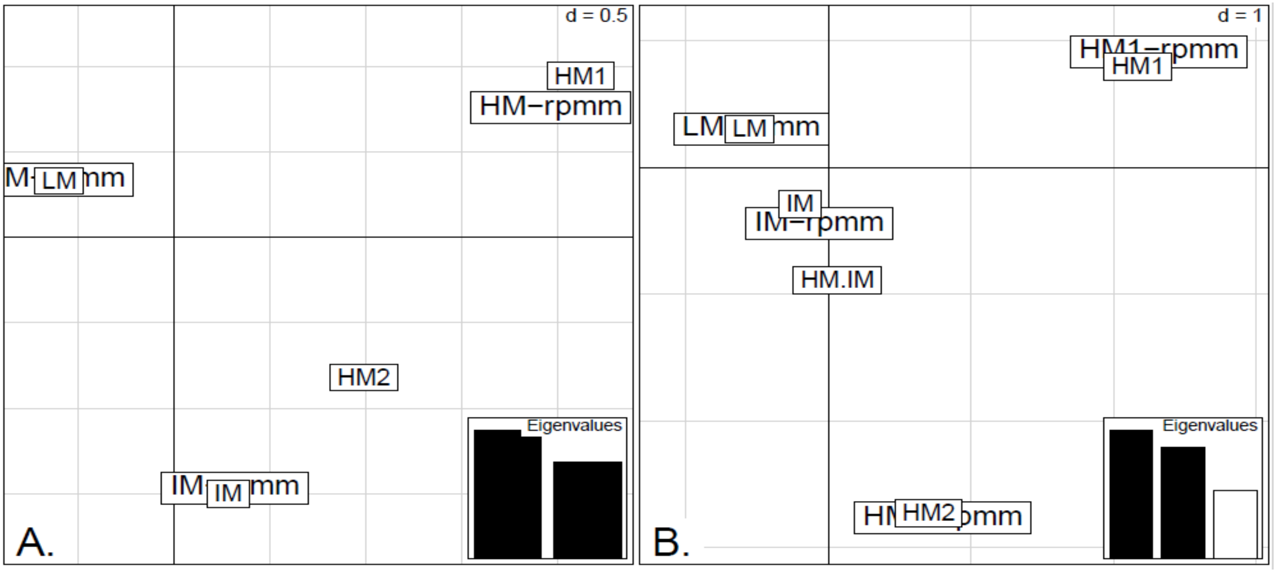

Figure S7. Factorial Correspondence Analysis (FCA) between the methylation clusters obtained with average linkage clustering (HM1, HM2, IM and LM) and the methylation clusters obtained with RPMM clustering (HM1-rpmm, HM2-rpmm, IM-rpmm and LM-rpmm) for (**A**) TCGA-Illu27; and (**B**) TCGA-Illu450 data sets.

Table S1. Dispersion Separability Criterion (DSC) computed for the COAD and READ entire datasets obtained with IlluHM27 and IlluHM450 platforms respectively. DSC is a ratio of between batch dispersion *vs.* within batch dispersion and is computed here in the plan formed by the first two principal components of each respective data set. Computations using the MBatch R library through the interface on the URL: http://bioinformatics.mdanderson.org/main/TCGABatchEffects:Overview. For all cases, DSC p-value < 0.0005.

TCGA Data Set	Platform	DSC (1, 2)
COAD	IlluHM27	0.405
READ	IlluHM27	0.138
COAD	IlluHM450	0.188
READ	IlluHM450	0.318

Table S2. Inflammatory (CRCA) and C2 (CCMS) Subtypes specific to the HM1 sub-cluster in TCGA-Illu27 and TCGA-Illu450 data sets (Figures 2 and 4).

HM1 Cluster Characteristics →	% of All Inflammatory	% of All C2	% Inflammatory & C2 Composition	Jaccard Similarity
Dataset ↓				
TCGA-Illu27	74	66	86	0.88: highly stable
TCGA-Illu450	50	61	69	0.73: borderline stable

Table S3. HM2 sub-cluster in TCGA-Illu27 and TCGA-Illu450 data sets; composition by Goblet-like (CRCA) and C3 (CCMS) Subtypes.

**HM2 Cluster Characteristics →**	**% of All Goblet-Like**	**% of All C3**	**% Goblet-Like & C3**	**Jaccard Similarity**
**Dataset ↓**				
TCGA-Illu27	25	32	73	0.65: fuzzy
TCGA-Illu450	40	46	48	0.78: stable

References

1. Sadanandam, A.; Lyssiotis, C.A.; Homicsko, K.; Collisson, E.A.; Gibb, W.J.; Wullschleger, S.; Ostos, L.C.; Lannon, W.A.; Grotzinger, C.; del Rio, M.; *et al.* A colorectal cancer classification system that associates cellular phenotype and responses to therapy. *Nat. Med.* **2013**, *19*, 619–625.
2. Marisa, L.; de Reyniès, A.; Duval, A.; Selves, J.; Gaub, M.P.; Vescovo, L.; Etienne-Grimaldi, M.C.; Schiappa, R.; Guenot, D.; Ayadi, M.; *et al.* Gene expression classification of colon cancer into molecular subtypes: Characterization, validation, and prognostic value. *PLoS Med.* **2013**, *10*, e1001453.
3. Isella, C.; Terrasi, A.; Bellomo, S.E.; Petti, C.; Galatola, G.; Muratore, A.; Mellano, A.; Senetta, R.; Cassenti, A.; Sonetto, C.; *et al.* Stromal contribution to the colorectal cancer transcriptome. *Nat. Genet.* **2015**, *47*, 312–319.
